# Shotgun and TMT-Labeled Proteomic Analysis of the Ovarian Proteins of an Insect Vector, *Aedes aegypti* (Diptera: Culicidae)

**DOI:** 10.1093/jisesa/ieac018

**Published:** 2022-03-18

**Authors:** Dawn L Geiser, Wenzhou Li, Daphne Q-D Pham, Vicki H Wysocki, Joy J Winzerling

**Affiliations:** 1 Nutritional Sciences, Division of Agriculture, Life and Veterinary Sciences, University of Arizona, Tucson, AZ 85721, USA; 2 Department of Chemistry and Biochemistry, College of Science, University of Arizona, Tucson, AZ 85721, USA; 3 Present Address: Amgen Incorporation, One Amgen Center Drive, Thousand Oaks, CA 91320, USA; 4 Department of Biological Sciences, University of Wisconsin-Parkside, Kenosha, WI 53141, USA; 5 Present Address: Department of Chemistry and Biochemistry, The Ohio State University, Columbus, OH 43210, USA

**Keywords:** bloodmeal, ferritin, iron, ovary, proteomics

## Abstract

*Aedes aegypti* [Linnaeus *in* Hasselquist; yellow fever mosquito] transmits several viruses that infect millions of people each year, including Zika, dengue, yellow fever, chikungunya, and West Nile. Pathogen transmission occurs during blood feeding. Only the females blood feed as they require a bloodmeal for oogenesis; in the bloodmeal, holo-transferrin and hemoglobin provide the females with a high iron load. We are interested in the effects of the bloodmeal on the expression of iron-associated proteins in oogenesis. Previous data showed that following digestion of a bloodmeal, ovarian iron concentrations doubles by 72 hr. We have used shotgun proteomics to identify proteins expressed in *Ae. aegypti* ovaries at two oogenesis developmental stages following blood feeding, and tandem mass tag-labeling proteomics to quantify proteins expressed at one stage following feeding of a controlled iron diet. Our findings provide the first report of mosquito ovarian protein expression in early and late oogenesis. We identify proteins differentially expressed in the two oogenesis development stages. We establish that metal-associated proteins play an important role in *Ae. aegypti* oogenesis and we identify new candidate proteins that might be involved in mosquito iron metabolism. Finally, this work identified a unique second ferritin light chain subunit, the first reported in any species. The shotgun proteomic data are available via ProteomeXchange with identifier PXD005893, while the tandem mass tag-labeled proteomic data are available with identifier PXD028242.

Mammalian iron metabolism involves numerous proteins, and is well characterized and complex (Byrne et al. 2013, Bogdan et al. 2016, Brissot and Loreal 2016, Dutkiewicz and Nowak 2018, Knutson 2019, Maio and Rouault 2020, Philpott et al. 2020, Liu et al. 2021, Plays et al. 2021). Of the multitude of proteins involved in this process, those found in mosquitoes have been recently reviewed by Whiten et al. (2017) and include only ferritin light chain 1 (LCH1), ferritin heavy chain (HCH), heme oxygenase, transferrin (Tf), Natural Resistance-Associated Macrophage Protein (NRAMP) (Duchemin and Paradkar 2017), iron regulatory protein1 (IRP1), the putative iron transporters, ZnT7, ZIP13, and ZIP11 (Tsujimoto et al. 2018), and dyspepsia (Tsujimoto et al. 2021). Yet, these insects receive a massive iron load in a bloodmeal, and of the iron absorbed from the meal, more than 50% is found in the developing ovaries and eggs (Zhou et al. 2007). In mammals, Tf transports iron in blood to the tissues, while ferritin is a cytoplasmic iron storage protein. However, in mosquitoes, ferritin serves both as a transporter and iron storage protein (Zhou et al. 2007, Geiser et al. 2009). In mammals, zinc, copper, and iron pathways often intersect, and several multicopper oxidases are required for iron metabolism (Vashchenko and MacGillivray 2013, Wang et al. 2018).

We are interested in identifying additional proteins involved in iron metabolism in mosquitoes. Since Zhou et al. (2007) demonstrated that a large portion of absorbed meal iron was delivered to the ovaries, we thought this tissue would be a good source for identifying proteins expressed following a bloodmeal. Such data also would broaden the understanding of mosquito oogenesis and development, and consequently, identify potential targets for insect control strategies. Before eclosion and mating, the ovaries of *Ae. aegypti* [Linnaeus *in* Hasselquist] enter the previtellogenic ‘resting’ phase when oocytes become competent to incorporate vitellogenin, and little follicle growth occurs. At the start of blood feeding, the initiation phase begins and lasts 3–10 hr, during which time follicle growth recommences and new microvilli and coated pits are observed on the surface of the oocyte (Clements 2000). Following the follicular growth re-initiation, the follicular epithelium separates allowing hemolymph contact with the oocyte, and incorporation of vitellogenin into yolk bodies. In the trophic phase, vitellogenin uptake increases linearly until 24 hr post bloodmeal (PBM); the basal lamina surrounding the follicular epithelium acts as a coarse filter permeable to lipids, polysaccharides, and proteins of up to 500 kDa or <11 nm, for incorporation into the oocyte. Passage of nutrients from the hemolymph into the oocyte concludes by 48 hr PBM, when deposition of an intact endochorionic layer between the oocyte and follicular epithelium ends the trophic phase. The oocyte grows to its final size and assumes its final form in the post-trophic phase with deposition of the exochorion by the follicular epithelium in preparation for fertilization and oviposition that occurs between 72 and 96 hr PBM.

Proteomics has proven valuable for evaluating the expression of large numbers of proteins from yeast (~4,000) to humans (>10,000) (Richards et al. 2015). Given the iron load of the bloodmeal, the transport of meal iron to the ovaries, and the requirement for iron during animal development (Gonzalez-Morales et al. 2015, Rivera-Perez et al. 2017), we employed two proteomics techniques, shotgun and tandem mass tag-labeling (TMT-labeling), to identify proteins present in this tissue following a bloodmeal. To our knowledge, this is the first report to identify proteins expressed at two different stages of oogenesis in *Aedes* ovaries PBM utilizing high throughput, high-resolution mass spectrometry. Shotgun proteomics identified several putative proteins involved in mosquito iron metabolism including a second ferritin light chain subunit (LCH2), the first identified in any species. We also employed TMT-labeling proteomics to quantify the expression of a subset of ovarian proteins in response to an iron meal.

## Materials and Methods

### Shotgun Proteomic Analysis

#### Mosquito Rearing

Mosquitoes were raised as noted elsewhere (Geiser et al. 2017); mated female mosquitoes were fed on Porcine blood eight to nine days post-eclosion. *Ae. aegypti* were a gift from Dr. Michael Riehle (Department of Entomology, University of Arizona).

#### Tissue Collection

In *Ae. aegypti*, blood feeding is required for oogenesis. We reasoned that we would identify the greatest number of proteins by analyzing samples during an early stage of oogenesis (the trophic phase, 24 hr PBM), and a very late stage (72 hr PBM) because, as noted, these times signify important gates in the development process (Anderson and Spielman 1971). Further, Akbari et al. (2013) reported that in a pair-wise comparison of the transcriptome at various times during egg development, every development stage was highly correlated with the adjacent stages with the exception of 36–48 and 60–72 hr, suggesting that these times represented developmental physiological transitions. To allow for biological variation, we blood fed animals on six different dates. For each date, we collected ovaries from 15 cold anesthetized animals at each developmental stage PBM (24, 72 hr, Supplement 1 [online only]); ovary pairs were dissected into disruption buffer (10 mM Tris-HCl, pH 7.9; 1.5 mM MgCl_2_; 0.5 mM DTT added fresh) with 2x Protease Inhibitor cocktail (Catalogue # 539131; EMD Millipore, Billerica, MA) added fresh. As ovaries were pooled from each time point, samples represented 6 different laboratory-raised *Ae. aegypti* mosquito populations and a total of 90 ovary pairs.

#### Sample Preparation and Ovary Proteome Analysis

Ovarian proteins were extracted as previously described elsewhere (Geiser et al. 2017), flash frozen in liquid nitrogen, and stored at –80°C. We found the mosquito ovary proteome dominated by a few high abundance proteins (e.g., vitellogenin), and thus, we used in-gel digestion rather than in-solution digestion. Samples for liquid chromatography–tandem mass spectrometry (LC-MS/MS) were thawed, and proteins from each time point were processed by the Analytical & Biological Mass Spectrometry Facility (University of Arizona, Tucson, AZ) as follows: 30 μg of ovary protein from each time point was boiled 20 min in Laemmli buffer (Laemmli 1970) containing 50 mM DTT as the reductant, separated via 12% SDS-PAGE, and stained with Bio-Safe Coomassie Blue (Catalogue # 1610786; Bio-Rad, Hercules, CA). Eight slices were cut from each gel lane according to molecular weight (MW) as demonstrated in Supplement 2 (online only): Slice 8, 200–150 kDa; Slice 7, 150–100 kDa; Slice 6, 100–70 kDa; Slice 5, 70–50 kDa; Slice 4, 50–35 kDa; Slice 3, 35–25 kDa; Slice 2, 25–17 kDa; Slice 1, 17–10 kDa. Proteins in each gel slice were trypsin digested overnight (1 µg trypsin (Catalogue # V511; Promega, Madison, WI): 5 µg sample protein; digestion conditions determined during protocol optimization) at 37°C, following the protocol (http://proteomics.arizona.edu/sites/proteomics.arizona.edu/files/In_gel_trypsin_digestion_May2012.pdf) provided by the Analytical & Biological Mass Spectrometry Facility (MassSpec@email.arizona.edu). Digested peptides were extracted from the individual gel pieces and stored separately at -20°C. Before analysis, individual gel band extracts were thawed and desalted on a C18 precolumn (100-μm id × 2 cm, Thermo Fisher Scientific) using the protocol provided by the Analytical & Biological Mass Spectrometry Facility (http://proteomics.arizona.edu/sites/proteomics.arizona.edu/files/SPE_C18_Clean_up_ZipTip.pdf). Desalted peptides (0.5 µg injected for each gel slice) were eluted as previously described by Chaw et al. (2015) via LTQ Orbitrap Velos mass spectrometer (Thermo Fisher Scientific, Waltham, MA) and an Advion nanomate ESI source (Advion, Ithaca, NY). Data-dependent scanning was performed as described by Jiang et al. (2014).

Peptide identification was performed using SeQuence IDentfication (SQID) (Li et al. 2011) against the *Ae. aegypti* database downloaded from the National Center for Biotechnology Information (NCBI; Genome ID: 44, downloaded on 23 June 2012) for the combined results for 8 gel pieces per time point. The search was performed with the forward database appended with a reverse database; the false discovery rate (FDR) was determined as FDR = 2 * ReverseID/(ForwardID + ReverseID). The *Ae. aegypti* forward and reverse database was used to establish criteria for protein identification. A maximum of 2 missed cleavages were allowed and methionine oxidation (M+16) and carbamidomethylation (C+57) were searched as variable modifications. The result spectra list for each time point was generated in Scaffold (Version 3.1.2; Proteome Software, Inc., Portland, OR) and ranked using a SQID score column to calculate peptide FDR. A 5% peptide FDR, a minimum of two unique peptides per protein, and a 1% protein FDR were used as the threshold for protein identification. Experimental results represent two time points each analyzed in duplicate. Running duplicates minimized the chances that a low abundance protein was unidentified at a time point due to sensitivity rather than a true difference between time points.

The mass spectrometry proteomics data have been deposited to the ProteomeXchange Consortium (Deutsch et al. 2020) via the PRIDE (Perez-Riverol et al. 2019) partner repository with the dataset identifier PXD005893 and 10.6019/PXD005893. Proteomic data include spectra, number of unique peptides identified, total number of peptides identified, and percent coverage for each protein identified in the following tables and analyses can be acquired from the original Scaffold file downloaded from the PRIDE Archive (https://www.ebi.ac.uk/pride/archive/) and viewed in the most recent version of Scaffold via free download from the Scaffold website (https://www.proteomesoftware.com/products/scaffold-5).

#### Criteria for Identifying Proteins at Each Development Stage

A candidate protein was validated as being only detected at either early or late development by meeting the following three criteria for duplicate analyses: (1) we identified a minimum of two unique peptides per protein; (2) all peptides had a greater than 95% identification confidence; and (3) peptides were only identified at one time point.

### TMT-Labeling Proteomic Analysis

#### Mosquito Rearing, Tissue Collection, and Sample Preparation

Since we anticipated identifying the greatest number of proteins from ovaries during the trophic phase of oogenesis (24 hr PBM), we chose to quantitate differences in ovary protein expression based on iron content of the diet at this developmental stage by labeling sample peptides using a tandem mass tag (TMT) Isobaric and Isotopic Mass Tagging kit. Mosquitoes were maintained as described above. Mated, female mosquitoes were fed one of two isoproteinic diets or a Porcine bloodmeal (Porcine BM, 603 ng Fe/μl) maintained at 27°C in glass feeders for 2 hr. The isoproteinic diets were: Kogan’s (1990) artificial bloodmeal (ABM) with hemoglobin ((+)Fe): 0.8% (w/v) Porcine Hemoglobin (Sigma, St. Louis, MO); 1.5% (w/v) Porcine IgG (Sigma); 10% (w/v) Porcine Albumin (Sigma); and 5 mM ATP (Sigma) in feeding buffer (~56 ng Fe/μl) and Kogan’s (1990) ABM without hemoglobin ((-)Fe): 10.7% (w/v) Porcine Albumin; 1.6% (w/v) Porcine IgG; and 5 mM ATP in feeding buffer (~24 ng Fe/μl). Tissues were collected as described above for early stage of oogenesis (24 hr PBM). Ovary proteins were extracted, and protein concentrations were determined as described above. Samples were flash frozen in liquid nitrogen and stored at −80°C until TMT-labeling.

#### Ovary Protein TMT-Labeling

Differences in ovary protein expression can be quantified by combining TMT-labeled peptides from ovaries of animals fed different diets and analyzing the peptides simultaneously by LC-MS/MS. Ovary samples at the trophic phase of oogenesis (24 hr PBM) for the (+)Fe diet, (-)Fe diet, and the Porcine BM were collected and processed as previously described and TMT-labeled following the manufacturer instructions for the TMT6plex kit (Catalogue # 90061; Thermo Fisher Scientific). Briefly, to prepare samples for TMT-labeling, 6 μl of each sample ((+)Fe: 33.9 μg; (-)Fe: 34.4 μg; Porcine BM: 41.4 μg) was diluted in sterile ddH_2_O up to 50 μl, then 45 μl 100 mM triethyl ammonium bicarbonate (TEAB) was added and mixed with each sample. Since these samples were complex protein mixtures, 5 μl 2% SDS was mixed in to bring the final volume to 100 μl. Tris(2-carboxyethyl)phosphine [TCEP (5 μl 200 mM)] was added to each sample, mixed, and incubated at 55°C for 1 hr in the dark. Further, 5 μl 375 mM iodoacetamide (IAA) was mixed into each sample and incubated at room temperature (RT) for 30 min in the dark. Finally, 1 ml acetone, prechilled at −20°C, was mixed with each sample and allowed to precipitate overnight at −20°C and then centrifuged 10 min at 8,000 × *g* at 4°C. Supernatants were decanted, and the precipitated pellets were dried at RT for 10 min. Sample pellets were suspended in 50 μl 100 mM TEAB, and trypsin digested in the solution overnight (1 µg trypsin (Promega):15 µg sample protein; digestion conditions determined during protocol optimization) at 37°C, following the protocol (https://proteomics.arizona.edu/sites/proteomics.arizona.edu/files/Solution_trypsin_digestion_2.pdf) provided by the Analytical & Biological Mass Spectrometry Facility (MassSpec@email.arizona.edu). TMT6plex labels were dissolved in 41 μl acetonitrile for 5 min, RT, with occasional vortexing, and 6 μg (based on original sample protein concentration) trypsin-digested sample was added to a unique TMT6plex label, and incubated for 1 hr, RT. Hydroxylamine (8 μl, 5%) mixed with each sample and incubated for 15 min, RT was used to quench the labeling reaction. Samples were dried down to pellets using a SpeedVac vacuum concentrator with medium heat. In preparation for LC-MS/MS, sample pellets were dissolved in 6 μl 0.1% trifluoroacetic acid (TFA) pH 3.0, yielding sample concentrations of 1 μg/μl (based on original sample protein concentration). Next, 2 μl of each TMT-labeled diet sample (Table 1) was combined into one tube for a total volume of 6 μl (Tube 1), which was then further divided into 2 tubes of 3 μl each (Tubes A and B). Each tube had a total protein concentration of 3 μg (based on original sample protein concentration). Samples were stored at −80°C until LC-MS/MS analysis.

**Table 1.** TMT-labeled sample preparation

Sample	TMT-label	Combined Samples	Tube 1 Divided	
(+)Fe	126	Tube 1 (6 μl)	Tube A (3 μl)	Tube B (3 μl)
(-)Fe	128			
Porcine BM	130			

(+)Fe = Artificial bloodmeal (ABM) with Iron; (-)Fe = ABM without Iron; Porcine BM = Porcine bloodmeal.

#### TMT-Labeled Ovary Proteome Analysis

Prior to analysis, each sample, Tube A or Tube B, was thawed and desalted using the protocol described: (http://proteomics.arizona.edu/sites/proteomics.arizona.edu/files/SPE_C18_Clean_up_ZipTip.pdf). Each TMT-labeled sample, Tube A or Tube B, was analyzed in triplicate by LC-MS/MS using equipment and methods described by Yuan et al. (2015); peptides on the analytical column were fractionated by holding at 5% solvent B (acetonitrile, 0.1% formic acid) for 10 min, followed by a solvent B gradient of 5–20% over 65 min, then by a solvent B gradient of 20-35 % over 45 min, then a 35–95% gradient of solvent B (0.1 min), ending with a solvent B (95%) hold for 5 min. Running multiple analyses of each tube minimizes the chances that a low abundance protein goes unidentified. Data dependent scanning was done as described in Jiang et al. (2014) with the following changes: the Orbitrap analyzer scanned *m*/*z* 350–1600 followed by collision-induced dissociation (CID) MS/MS of the ten most intense ions in the linear ion trap analyzer, and the dynamic exclusion was set on an exclusion list for 30 s after a single MS/MS.

The raw file of the MS/MS spectra output and the spectra were searched using Proteome Discoverer version 2.4.0.305 software (Thermo) for quantitative analysis against the *Ae. aegypti* database downloaded from UniProt (Aedesaegypti_UniprotKB_2020_7159.fasta). For protein identification, the following options were used: MS/MS spectra matches reflected fully tryptic peptides with up to 2 missed cleavage sites, variable modifications considered during the search included methionine oxidation (15.995 Da), TMT6plex on the N termini (+229) and cysteine carbamidomethylation. Proteins were identified and validated according to Yuan et al. (2015) and the results displayed with Scaffold Q+S v 4.11.0 (Proteome Software, Inc.); TMT 6 plex (N-term) and TMT 6 plex (K). Variable modifications included Met residue oxidation (M) and filtering at an FDR ≤ 0.01; for quantification, the median of only unique peptides of the protein are used to calculate the protein ratios, and for experimental bias, all peptide ratios were normalized by the median protein ratio, and the median protein ratio should be 1 after normalization.

The mass spectrometry proteomics data have been deposited to the ProteomeXchange Consortium (Deutsch et al. 2020) via the PRIDE (Perez-Riverol et al. 2019) partner repository with the dataset identifier PXD028242 and 10.6019/PXD028242. Proteomic data include spectra, number of unique peptides identified, total number of peptides identified, and percent coverage for each protein identified in the following tables and analyses can be acquired from the original Scaffold and Proteome Discoverer files downloaded from the PRIDE Archive (https://www.ebi.ac.uk/pride/archive/). The Scaffold files can be viewed in the most recent version of Scaffold Q+S via free download from the Scaffold website (https://www.proteomesoftware.com/products/scaffold-quant). The Proteome Discoverer files can be opened in the most recent version of Proteome Discoverer via free trial download from the ThermoFisher Scientific website (https://www.thermofisher.com/us/en/home/industrial/mass-spectrometry/liquid-chromatography-mass-spectrometry-lc-ms/lc-ms-software/multi-omics-data-analysis/proteome-discoverer-software.html).

#### Criteria for Identifying Proteins Responsive to Dietary Iron

A candidate protein was validated as being regulated by iron in the diet during the trophic phase of oogenesis by meeting the following criteria for multiple analyses: (1) a minimum of two unique peptides per protein were identified; (2) all peptides had a greater than 95% identification confidence; (3) protein was identified in both the Proteome Discoverer and Scaffold outputs from the same analysis; (4) protein was identified in two separate analyses; (5) the difference between increased or decreased expression of a protein due dietary iron was significant (*p* < 0.05) using the Holm-Sidak method for multiple unpaired *t*-tests in Prism 6, Version 6.07 (Graph Pad Software, Inc., San Diego, CA); and (6) the direction of protein expression, either upregulated or downregulated, was consistent among analyses.

#### Bioinformatic Analysis of the Ovary Proteome and TMT-Labeled Ovary Proteome

Scaffold or Proteome Discoverer identified protein GenBank GI numbers were analyzed for their correct unique identifiers using Batch Entrez (Coordinators 2014). Those identifiers that were found to be an old version were updated with a new GI number, while those identifiers that were found to be eliminated were further analyzed by BLASTP (Coordinators 2014) to obtain the correct GI Number. UniProtKB (UniProt 2015) analysis was performed using the corrected GI numbers; entry information for each identified protein was obtained (UniProtKB ID: Status, Protein names, Gene names, and Length; Gene Ontology (GO): Biological Process, Molecular Function, and Cellular Component; GO Identifiers: Developmental Stage, Induction, Tissue Specificity, Pathway, Subcellular Location, Protein Families, Metal Binding, PubMed ID, Cross-reference (STRING), and Cross-reference [InterPro]). Duplicate protein entries (the same protein identified with separate GI numbers) are acknowledged in supplementary tables in italics. The Database for Annotation, Visualization, and Integrated Discovery (DAVID version 6.7 [Huang da et al. 2009a,b]) was used to analyze the Gene names obtained from the UniProtKB analysis; DAVID identifiers were recorded and the proteomic data set was annotated and grouped by GO terms for biological process, molecular function, and cellular component. Proteins that were not in the DAVID database in all pertinent tables and supplementary data are identified in bold-faced font. Proteins found in the DAVID database were further analyzed using functional annotation clustering with a minimum *p* < 0.05 to identify enrichment of GO categories against the *Ae. aegypti* genomic background.

## Results and Discussion

### Characteristics of the Aedes PBM Ovary Proteome

**Fig. 1.**
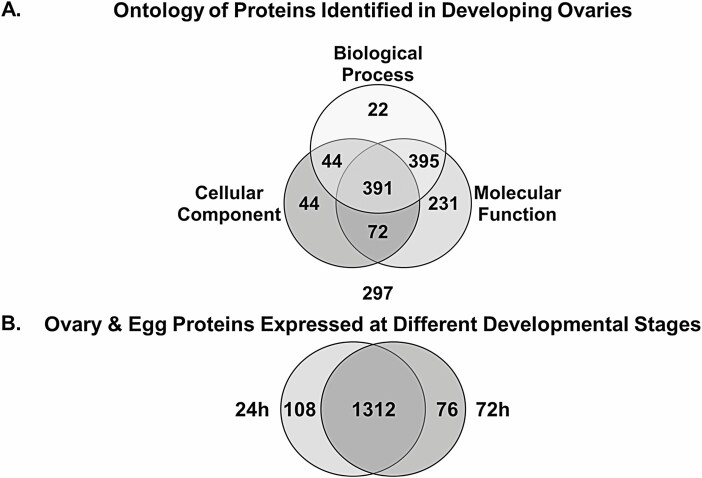
**A. Ontology of *Ae. aegypti* Ovary Proteins Identified in Developing Ovaries.** Scaffold identified proteins were categorized via general gene ontology (GO) terms (e.g., Biological Process, Cellular Component, and Molecular Function) obtained from NCBI, UniProt, and DAVID databases as described in the Materials and Methods. There were 297 proteins that could not be categorized using general GO terms. **B. Ovary and Egg Proteins Expressed at Different Developmental Stages.** Proteins detected 24 and 72 hr post bloodmeal (PBM) were identified using the stringency criteria described in the Materials and Methods. Of 1,496 proteins identified using Scaffold, 108 proteins were detected only at 24 hr PBM, while 76 proteins were detected only at 72 hr PBM.

We used shotgun proteomics to identify proteins present in two developmental stages; if a protein that met the criteria noted was present in both replicates, it was identified at that developmental stage. Data from Scaffold analyses identified 1,525 proteins of which 29 were duplicates providing a database of 1,496 *unique* proteins. The *Ae. aegypti* ovary proteome database with ontology analysis using general GO terms from the NCBI, UniProtKB, and DAVID databases, is available in Supplement 3 (online only). Of the 1,496 proteins, 1,199 were assigned an ontology category (Fig. 1A, Table 2). Many proteins identified to more than one functional term as shown in the Venn diagram (Fig. 1A). General GO categories and specific GO terms used in our analysis only indicate likely gene functions in processes for the GO term name, and do not provide definitive function without further empirical research. The numbers and percentages of proteins that are sorted to various pathways or processes are shown in Table 3. We found 242 proteins (16%) involved with ATP and GTP processes indicating that development provokes a high demand for energy. Of the 185 ATP-associated proteins, 12% are part of the proton- or ion-transporting mechanism (22/185) and 84% (156/185) are involved in ATP-binding. Among ATP-binding proteins, 16% are involved in phosphatase/kinase activities (25/156), 6% in stress response (9/156), and 6% are involved in DNA replication (10/156). There are 60 GTP-associated proteins and 93% (56/60) are involved in GTP-binding. Of the GTP-binding proteins, 42% are active in signal transduction (24/56), 9% in catabolism (5/56), and 13% with cytoskeletal motor activity (7/56). About 7% of the ATP- or GTP-binding proteins play a role in catabolic process and 6% in cytoskeleton motor activity (12/211). In addition to the ATP-associated proteins involved in DNA replication, as we might expect there is strong response for proteins involved in cellular multiplication and transcription, 5% as nucleic acid binding proteins (72/1496), 4% as DNA processing proteins (61/1496), and 2% as proteins of DNA transcription (2%, 23/1496) that includes those engaged in DNA binding (35%, 8/23) and polymerase activity (35%, 8/23).

**Table 2.** Gene ontology terms for *Ae. aegypti* ovary proteins

GO Term	# of Proteins	% of Total Proteins
Biological Process	852	57.0
Molecular Function	1,089	72.8
Cellular Component	551	36.8

GO = Gene ontology (http://www.ncbi.nlm.nih.gov/, http://www.uniprot.org/, https://david.ncifcrf.gov/); % of Total Proteins = # of Proteins/1496.

**Table 3.** Gene ontology categories of *Ae. aegypti* ovary proteins

GO Category	# of Proteins	% of Total Proteins
ATP/GTP processes	242	16
Metal-Associated Proteins	171	11
Transport	146	10
Translation	123	8
Proteolysis	82	5
Nucleic Acid Binding	72	5
DNA Processing	61	4
Protein Modification	58	4
Odorant Binding	39	3
Redox Proteins	35	2
Carbohydrate Metabolism	35	2
Lipid Metabolism	35	2
Transcription	23	2
Reproduction/Development	19	1
Glucose Metabolism	17	1

GO = Gene ontology (http://www.ncbi.nlm.nih.gov/, http://www.uniprot.org/, https://david.ncifcrf.gov/); % of Total Proteins = # of Proteins/1496.

Accompanying the strong energy demands of development is a substantial investment in protein biogenesis, processing, and turnover. Of proteins involved in translation (8%, 123/1496), 20% are linked with translation initiation (25/123), and 13% with RNA binding (16/123). Female mosquitoes also invested 4% (58/1496) of proteins in protein modification with 50% of the proteins involved in protein folding (29/58), 22% as kinases and phosphatases (13/58), 5% as glycotransferases (3/58), and 7% involved with lipid addition (4/58). Further, proteolytic enzymes are 5% (82/1496) of the proteome. Proteolytic enzymes expressed in this tissue include: 23 serine (endo)peptidases, 14 threonine (endo)peptidases, 13 cysteine (endo)peptidases, 2 dipeptidases, 1 aspartic peptidase, and 7 other catabolic proteases. We think it likely this reflects processing of proteins by the ovaries with recovery of amino acids to synthesize new proteins required for egg development.

The ovary PBM proteome contains 10% (146/1496) transport proteins that include proteins involved in both vesicle (20/146) and intracellular transport (40/146). Among the vesicular transporters, the clathrin and COP vesicular transport constitutes the largest aggregate (10/20). The COP-II vesicle pathway is important for iron metabolism as our previous work indicates that ferritin is secreted from mosquito cells using this pathway (Geiser et al. 2009).

Blood feeding induces oxidation reactions in the mosquito (Lima et al. 2012, Bottino-Rojas et al. 2015). To handle the redox toxicity of a bloodmeal, *Ae. aegypti* dedicate ~2% (35/1496) of the identified proteins for oxidation-reduction processes with the largest component as oxidoreductases (49%,17/35). This also could reflect the engagement of these proteins in the movement of electrons and electron capture.

Female mosquitoes seek a specific host for blood feeding. About 3% (39/1496) of the ovary and egg proteome is invested in odorant binding proteins known for their involvement in host preference detection (International Glossina Genome 2014). Akbari, et al. (2013) found the ovarian transcriptome enriched in odorant transcripts during oogenesis. Odorant proteins have been observed in *Drosophila* male reproductive organs and could be transferred to females during mating. Odorant proteins were also found in *Aedes* eggshell and *Anopheles* eggs (Yamamoto and Takemori 2010, Sirot et al. 2011, Mastrobuoni et al. 2013, Marinotti et al. 2014).

#### Development Stage-Specific Ovary Proteins

Akbari, et al., (2013) found that gene expression was upregulated 5-fold in the ovary PBM and that the transcriptome signature differed during oogenesis. Thus, as expected, some ovary proteins in the current study are differentially expressed at each developmental stage:108 proteins that were detected only at the early developmental stage, while 76 proteins were expressed only at the later stage (Fig. 1B, Tables 4 and 5, respectively. Although many of these proteins are found in organisms from other phylogenetic lineages (conserved hypothetical proteins), they remain functionally uncharacterized in *Ae. aegypti* (Galperin and Koonin 2004), while others are predicted from an open reading frame (hypothetical proteins) with no experimental evidence of translation (Desler et al. 2009). These data offer initial proof that the hypothetical proteins are expressed. Supplement 4 (online only) lists early (24 hr) and late (72 hr) stage-specific ovary proteins obtained from DAVID functional annotation cluster analysis that provides gene enrichment information against the background of the whole *Ae. aegypti* genome. According to Huang et al. (2009a, b), the DAVID gene enrichment analysis highlights the most over-represented (enriched) biological annotations out of thousands of linked terms and contents found in a genomic background. While the DAVID results demonstrate the enrichment of some proteins compared to the whole *Aedes* database, it does not account for all the proteins identified in our analysis. Thus, we provide this analysis as supplementary data, and we report protein numbers/category rather than DAVID enrichment.

**Table 4.** *Ae. aegypti* ovary proteins expressed in early development post bloodmeal (24 hr)

Gene Name	Protein Name	GI Number^*a*^	UniProtKB ID	DAVID IDs
AaeL_AAEL012318	2-Amino-3-ketobutyrate coenzyme a ligase	108871293	Q16MH0_AEDAE	2093859
AaeL_AAEL006684	3-Hydroxyisobutyrate dehydrogenase	108877486	GLYR1_AEDAE	2104787
AaeL_AAEL012681	60S ribosomal protein L24	108870892	Q16LE3_AEDAE	2103461
AaeL_AAEL006307	Acetyl-coa carboxylase	108877881	Q176P0_AEDAE	2091871
AaeL_AAEL012731	Adenylate kinase 1, putative	108870854	KAD2_AEDAE	2112765
AaeL_AAEL005477	Amidase	108878831	Q179X9_AEDAE	2097451
AaeL_AAEL012621	Arginine/serine-rich splicing factor	108870967	Q16LJ8_AEDAE	2101940
AaeL_AAEL006577	Aspartyl-tRNA synthetase	108877614	Q175Q3_AEDAE	2097261
AaeL_AAEL004297	ATP-citrate synthase	108880117	Q17D87_AEDAE	2087935
AaeL_AAEL004859	ATP-dependent RNA helicase	108879498	Q0IFJ1_AEDAE	2102674
AaeL_AAEL012421	Cadherin	108871179	Q16M52_AEDAE	2096127
AaeL_AAEL009392	Chromosome region maintenance protein 5/exportin	108874516	Q16VX2_AEDAE	2113917
AaeL_AAEL011673	cop9 signalosome complex subunit	108872010	Q1DH49_AEDAE	2106421
AaeL_AAEL001308	CRAL/TRIO domain-containing protein	108883381	Q17LK7_AEDAE	2098584
AaeL_AAEL005455	ctp synthase	108878841	Q17A05_AEDAE	2098190
AaeL_AAEL008468	Cysteine synthase	108875525	Q16YQ8_AEDAE	2111660
AaeL_AAEL004054	Cytochrome P450	108880360	Q17DS9_AEDAE	2116061
AaeL_AAEL014414	DEAD box ATP-dependent RNA helicase	108869079	Q16GE2_AEDAE	2106563
AaeL_AAEL010787	DEAD box ATP-dependent RNA helicase	108872968	Q16RY3_AEDAE	2086759
AaeL_AAEL010317	DEAD box ATP-dependent RNA helicase	108873494	Q0IEJ1_AEDAE	2105052
AaeL_AAEL004017	DNA polymerase v	108880403	Q17DX3_AEDAE	2088068
AaeL_AAEL000440	DNA repair/transcription protein met18/mms19	108884288	Q17PB8_AEDAE	2083068
AaeL_AAEL012584	DNA topoisomerase/gyrase	108871009	Q0IEA2_AEDAE	2114318
AaeL_AAEL006012	Factor for adipocyte differentiation	108878240	Q177W1_AEDAE	2090808
AaeL_AAEL007042	Far upstream (fuse) binding protein	108877074	Q173N9_AEDAE	2086496
AaeL_AAEL001194	Fatty acid synthase	108883503	Q17M16_AEDAE	2105543
AaeL_AAEL000693	fkbp-rapamycin associated protein	108884015	Q17NH6_AEDAE	2104556
AaeL_AAEL007080	Glycerol-3-phosphate acyltransferase	108877043	Q173M5_AEDAE	2110913
AaeL_AAEL011255	GTP-binding protein-invertebrate	108872457	Q16QL1_AEDAE	2086215
AaeL_AAEL009067	Helicase	108874885	Q16WX8_AEDAE	2103071
AaeL_AAEL002048	Histidyl-tRNA synthetase	108882557	Q17JC2_AEDAE	2099602
AaeL_AAEL004586	Histone deacetylase	108879776	Q17CF0_AEDAE	2097921
AaeL_AAEL011657	Importin alpha	108872016	Q16PF9_AEDAE	2088750
AaeL_AAEL014847	Innexin	108868690	Q16FA2_AEDAE	2114815
AaeL_AAEL003193	Inorganic pyrophosphatase	108881298	Q17G61_AEDAE	2097281
AaeL_AAEL009273	Inosine-5-monophosphate dehydrogenase	108874647	Q16WB3_AEDAE	2102535
AaeL_AAEL008542	Kinesin heavy chain subunit	108875436	Q16YJ5_AEDAE	2102578
AaeL_AAEL009932	Lethal giant larva, putative	108873918	Q16UF4_AEDAE	2095370
AaeL_AAEL012251	Low-density lipoprotein receptor (ldl)	108871366	Q16MM6_AEDAE	2083611
AaeL_AAEL004134	Lupus la ribonucleoprotein	108880294	Q17DI5_AEDAE	2085456
AaeL_AAEL011978	Mannosidase alpha class 2a	108871676	Q16NG2_AEDAE	2083017
AaeL_AAEL001279	Merozoite surface protein, putative	108883434	Q16VW0_AEDAE	2097178
AaeL_AAEL006085	Methylenetetrahydrofolate dehydrogenase	108878146	Q177P7_AEDAE	2089050
AaeL_AAEL006917	MG-160, putative	108877209	Q174F8_AEDAE	2111877
AaeL_AAEL001605	Microtubule binding protein, putative	108883027	Q17KQ2_AEDAE	2108479
AaeL_AAEL002834	Myo-inositol-1 phosphate synthase	108881692	Q17H06_AEDAE	2088405
AaeL_AAEL007439	Myosin light chain 1, putative	108876630	Q172D1_AEDAE	2098541
AaeL_AAEL009706	ns1 binding protein	108874167	Q16V35_AEDAE	2100430
AaeL_AAEL009859	Nucleolar GTP-binding protein	108874003	Q16UL5_AEDAE	2109222
AaeL_AAEL005567	Nucleosome assembly protein	108878690	Q1HR22_AEDAE	2103661
AaeL_AAEL004613	Phenylalanyl-tRNA synthetase beta chain	108879755	Q17C95_AEDAE	2085650
AaeL_AAEL013227	PIWI	108870315	Q16JS1_AEDAE	2107620
AaeL_AAEL004484	Predicted protein	108879889	Q0IFM9_AEDAE	2085963
AaeL_AAEL008099	Procollagen-lysine,2-oxoglutarate 5-dioxygenase	108875910	Q0IER9_AEDAE	2094725
AaeL_AAEL013431	Proline oxidase	108870085	Q16J67_AEDAE	2083345
AaeL_AAEL003595	Protein serine/threonine kinase, putative	108880898	Q17F09_AEDAE	2102240
AaeL_AAEL007799	Regulator of chromosome condensation	108876236	Q170U4_AEDAE	2108247
AaeL_AAEL001397	Ribonuclease	108883257	Q17L93_AEDAE	2096583
AaeL_AAEL011089	Ribonucleoprotein	108872624	Q16R25_AEDAE	2084158
AaeL_AAEL011282	Ribosomal RNA small subunit methyltransferase b (sun)	108872435	Q16QI0_AEDAE	2107390
AaeL_AAEL001169	Ribosome biogenesis protein bop1 (block of proliferation 1 protein)	108883527	BOP1_AEDAE	2101661
AaeL_AAEL012171	RNA recognition motif protein split ends	108871456	Q16MW3_AEDAE	2088301
AaeL_AAEL001352	Scaffold attachment factor b	108883320	Q17LJ1_AEDAE	2098879
AaeL_AAEL010344	SEC14, putative	108873476	Q16T89_AEDAE	2095525
AaeL_AAEL007382	Serine-threonine kinase receptor-associated protein (strap)	108876724	Q1HQK6_AEDAE	2098255
AaeL_AAEL012401	Short-chain dehydrogenase	108871205	Q16M73_AEDAE	2100938
AaeL_AAEL005856	Signal recognition particle receptor alpha subunit (sr-alpha)	108878422	Q178J5_AEDAE	2085373
AaeL_AAEL008719	Sm protein G, putative	108875262	Q16XZ1_AEDAE	2086931
AaeL_AAEL005655	Sorting nexin	108878632	Q179E9_AEDAE	2085779
AaeL_AAEL003758	Sorting nexin	108880687	Q0IG11_AEDAE	2115044
AaeL_AAEL003443	Threonine dehydrogenase	108881080	Q17FE0_AEDAE	2092662
AaeL_AAEL002848	Tubulin beta chain	108881721	Q17GX8_AEDAE	2089138
AaeL_AAEL007160	Ubiquilin 1,2	108876959	Q173F1_AEDAE	2105399
AaeL_AAEL006910	Ubiquitination factor E4	108877215	Q174F4_AEDAE	2104342
AaeL_AAEL005930	Ubiquitin-protein ligase	108878317	Q178F7_AEDAE	2090371
AaeL_AAEL000444	UDP-glucose glycoprotein:glucosyltransferase	108884277	Q17PC9_AEDAE	2104456
AaeL_AAEL009099	Uridine cytidine kinase i	108874851	Q16WT5_AEDAE	2090772
AaeL_AAEL006516	Vacuolar ATP synthase subunit h	108877664	Q0IF50_AEDAE	2083704
AaeL_AAEL010584	Vesicular mannose-binding lectin	108873193	Q16SI7_AEDAE	2085013
AaeL_AAEL001616	Vesicular-fusion protein nsf	108883051	Q17KM8_AEDAE	2110780
AaeL_AAEL011535	wd-repeat protein	108872149	Q16EG4_AEDAE	2103309
AaeL_AAEL002784	Zinc finger protein	108881741	Q17H47_AEDAE	2104658
AaeL_AAEL014180	Conserved hypothetical protein	108869315	Q16H24_AEDAE	2085234
AaeL_AAEL013968	Conserved hypothetical protein	108869539	Q16HN8_AEDAE	2098860
AaeL_AAEL013334	Conserved hypothetical protein	108870197	Q16JH2_AEDAE	2090591
AaeL_AAEL013025	Conserved hypothetical protein	108870541	Q16KE3_AEDAE	2112497
AaeL_AAEL012980	Conserved hypothetical protein	108870590	PESC_AEDAE	2099663
AaeL_AAEL012618	Conserved hypothetical protein	108870976	Q16LK7_AEDAE	2106207
AaeL_AAEL012325	Conserved hypothetical protein	108871287	Q16ME7_AEDAE	2111115
AaeL_AAEL012243	Conserved hypothetical protein	108871375	Q16MP0_AEDAE	2096351
*AaeL_AAEL012243*	*Conserved hypothetical protein*	*108871376*	*Q16MN9_AEDAE*	
AaeL_AAEL011960	Conserved hypothetical protein	108871682	Q16NI7_AEDAE	2095194
AaeL_AAEL007647	Conserved hypothetical protein	108876414	Q171H7_AEDAE	2115477
AaeL_AAEL005277	Conserved hypothetical protein	108879056	Q17AL0_AEDAE	2103462
AaeL_AAEL004699	Conserved hypothetical protein	108879669	Q0IFK9_AEDAE	2102389
AaeL_AAEL004471	Conserved hypothetical protein	108879920	Q17CQ1_AEDAE	2087759
AaeL_AAEL003949	Conserved hypothetical protein	108880487	Q17E26_AEDAE	2088919
AaeL_AAEL002387	Conserved hypothetical protein	108882206	Q17IE6_AEDAE	2091027
AaeL_AAEL001448	Conserved hypothetical protein	108883195	Q17L75_AEDAE	2086517
AaeL_AAEL001129	Conserved hypothetical protein	108883537	Q17M82_AEDAE	2108360
AaeL_AAEL000695	Conserved hypothetical protein	108884042	Q17NF1_AEDAE	2085746
AaeL_AAEL002036	Hypothetical protein AaeL_AAEL002036	108882647	Q17JG8_AEDAE	2105722
AaeL_AAEL003671	Hypothetical protein AaeL_AAEL003671	108880767	Q17EV9_AEDAE	2094903
AaeL_AAEL004068	Hypothetical protein AaeL_AAEL004068	108880362	Q17DS7_AEDAE	2109814
AaeL_AAEL006751	Hypothetical protein AaeL_AAEL006751	108877414	Q174Z2_AEDAE	2096216
AaeL_AAEL007182	Hypothetical protein AaeL_AAEL007182	108876932	Q173D0_AEDAE	2086366
AaeL_AAEL010899	Hypothetical protein AaeL_AAEL010899	108872845	Q16RP2_AEDAE	2092031
AaeL_AAEL012018	Hypothetical protein AaeL_AAEL012018	108871627	Q16NC2_AEDAE	2110496
AaeL_AAEL012022	Hypothetical protein AaeL_AAEL012022	108871626	Q16NC3_AEDAE	2108627

^*a*^Corrected; UniProtKB ID = UniProt (http://www.uniprot.org/); DAVID ID = DAVID (https://david.ncifcrf.gov/).

Gene name = VectorBase (https://www.vectorbase.org/); Protein Name = NCBI; GI Number = NCBI (http://www.ncbi.nlm.nih.gov/); *Duplicate*.

**Table 5.** *Ae. aegypti* ovary proteins expressed in late development post bloodmeal (72 hr)

Gene Name	Protein Name	GI Number^*a*^	UniProtKB ID	DAVID IDs
AaeL_AAEL007097	4-Nitrophenylphosphatase	108877030	Q0IF18_AEDAE	2104858
AaeL_AAEL004739	Acyl-coa dehydrogenase	108879621	Q17BX4_AEDAE	2094528
AaeL_AAEL000931	Alkaline phosphatase	108883751	Q1HQK7_AEDAE	2110210
AaeL_AAEL000889	Carboxylesterase	403182383	Q17MW9_AEDAE	2096875
AaeL_AAEL006389	Cathepsin L	91992512	Q1PA54_AEDAE	2107437
AaeL_AAEL000375	Cysteine-rich venom protein, putative	108884380	Q17PL2_AEDAE	2112832
AaeL_AAEL000317	Cysteine-rich venom protein, putative	108884384	Q17PK8_AEDAE	2091681
AaeL_AAEL002046	Cytochrome P450	108882552	Q17JC7_AEDAE	2115184
AaeL_AAEL005325	Dopachrome-conversion enzyme (DCE) isoenzyme, putative	108879005	Q17AD1_AEDAE	2105615
**AaeL_AAEL017403**	***Drosophila* melanogaster vitelline membrane protein homolog**	**265212**	**V15A2_AEDAE**	
AaeL_AAEL009838	Glycogen debranching enzyme	108874010	Q16UP5_AEDAE	2100433
AaeL_AAEL003094	Glycoprotein, putative	108881423	Q17GD8_AEDAE	2089364
AaeL_AAEL010506	GTP-binding protein alpha subunit, gna	108873287	Q16SS3_AEDAE	2094613
AaeL_AAEL012960	Importin alpha	108870611	Q16KJ6_AEDAE	2102342
AaeL_AAEL015590	Juvenile hormone esterase	403183494	Q1DGL0_AEDAE	2084956
AaeL_AAEL005182	Juvenile hormone esterase	403182693	Q17AV4_AEDAE	2090333
AaeL_AAEL005198	Juvenile hormone esterase	108879131	Q17AV2_AEDAE	2083411
AaeL_AAEL004401	Peroxinectin	403182638	Q17CY9_AEDAE	2107800
AaeL_AAEL003612	Peroxinectin	108880850	Q17EY4_AEDAE	2107934
AaeL_AAEL013492	Prophenoloxidase	108870017	Q16IZ1_AEDAE	2103417
AaeL_AAEL011763	Prophenoloxidase	108871901	Q16P46_AEDAE	2083146
AaeL_AAEL006830	Yellow protein precursor	108877328	Q174P5_AEDAE	2107792
AaeL_AAEL014876	Conserved hypothetical protein	108868662	Q16F71_AEDAE	2096795
AaeL_AAEL014430	Conserved hypothetical protein	108869062	Q16GC2_AEDAE	2088320
AaeL_AAEL014431	Conserved hypothetical protein	108869063	Q16GC1_AEDAE	2107489
AaeL_AAEL013720	Conserved hypothetical protein	108869792	Q16ID1_AEDAE	2094831
AaeL_AAEL013719	Conserved hypothetical protein	108869793	Q16ID0_AEDAE	2096276
AaeL_AAEL013569	Conserved hypothetical protein	108869942	Q16IS0_AEDAE	2089562
AaeL_AAEL011238	Conserved hypothetical protein	108872476	Q16QL6_AEDAE	2090398
AaeL_AAEL010874	Conserved hypothetical protein	108872868	Q16RQ8_AEDAE	2094827
AaeL_AAEL010872	Conserved hypothetical protein	108872870	Q16RQ6_AEDAE	2103307
AaeL_AAEL010848	Conserved hypothetical protein	108872920	Q16RT9_AEDAE	2085186
AaeL_AAEL010718	Conserved hypothetical protein	108873051	Q16S35_AEDAE	2105962
AaeL_AAEL010714	Conserved hypothetical protein	108873052	Q16S34_AEDAE	2114747
AaeL_AAEL009433	Conserved hypothetical protein	108874461	Q16F73_AEDAE	2094820
AaeL_AAEL008640	Conserved hypothetical protein	108875341	Q16Y73_AEDAE	2105793
AaeL_AAEL007339	Conserved hypothetical protein	108876745	Q172N7_AEDAE	2088253
AaeL_AAEL007115	Conserved hypothetical protein	108876998	Q173K6_AEDAE	2087236
AaeL_AAEL007096	Conserved hypothetical protein	108877015	Q0IF33_AEDAE	2100673
AaeL_AAEL006387	Conserved hypothetical protein	108877806	Q176E5_AEDAE	2103143
AaeL_AAEL006396	Conserved hypothetical protein	108877807	Q176E4_AEDAE	2087992
AaeL_AAEL006385	Conserved hypothetical protein	108877809	Q176E2_AEDAE	2087347
AaeL_AAEL006328	Conserved hypothetical protein	108877877	Q176I1_AEDAE	2087316
AaeL_AAEL006336	Conserved hypothetical protein	108877878	Q176I0_AEDAE	2087698
AaeL_AAEL006143	Conserved hypothetical protein	108878096	Q177F6_AEDAE	2111130
AaeL_AAEL005756	Conserved hypothetical protein	108878508	Q178W6_AEDAE	2102962
AaeL_AAEL005759	Conserved hypothetical protein	108878509	Q178W5_AEDAE	2098955
AaeL_AAEL004856	Conserved hypothetical protein	108879531	Q0IFG0_AEDAE	2112549
AaeL_AAEL003525	Conserved hypothetical protein	108880947	Q0IG39_AEDAE	2101303
AaeL_AAEL003513	Conserved hypothetical protein	108880948	Q0IG38_AEDAE	2112828
AaeL_AAEL003511	Conserved hypothetical protein	108880950	Q0IG36_AEDAE	2085395
AaeL_AAEL003311	Conserved hypothetical protein	108881181	Q17FU5_AEDAE	2107585
AaeL_AAEL002333	Conserved hypothetical protein	108882281	Q17IG3_AEDAE	2096625
AaeL_AAEL001487	Conserved hypothetical protein	108883165	Q17L36_AEDAE	2115550
AaeL_AAEL001153	Conserved hypothetical protein	108883522	Q17LZ7_AEDAE	2115993
AaeL_AAEL001189	Conserved hypothetical protein	108883523	Q17LZ6_AEDAE	2087956
AaeL_AAEL000821	Conserved hypothetical protein	108883902	Q17N18_AEDAE	2094726
AaeL_AAEL000835	Conserved hypothetical protein	108883908	Q17N12_AEDAE	2114469
AaeL_AAEL000833	Conserved hypothetical protein	108883911	Q17N09_AEDAE	2084704
AaeL_AAEL000837	Conserved hypothetical protein	108883912	Q17N08_AEDAE	2090646
AaeL_AAEL000827	Conserved hypothetical protein	108883914	Q17N06_AEDAE	2087349
AaeL_AAEL000507	Conserved hypothetical protein	108884221	Q17P51_AEDAE	2090052
AaeL_AAEL000496	Conserved hypothetical protein	108884222	Q17P50_AEDAE	2093466
AaeL_AAEL000344	Conserved hypothetical protein	108884432	Q17PG0_AEDAE	2101527
AaeL_AAEL000377	Conserved hypothetical protein	108884433	Q17PF9_AEDAE	2087024
AaeL_AAEL000350	Conserved hypothetical protein	108884436	Q17PF5_AEDAE	2085195
AaeL_AAEL000144	Conserved hypothetical protein	108884628	Q17Q63_AEDAE	2083441
AaeL_AAEL000319	Hypothetical protein AaeL_AAEL000319	108884434	Q17PF7_AEDAE	2104507
AaeL_AAEL001174	Hypothetical protein AaeL_AAEL001174	108883524	Q17LZ5_AEDAE	2092531
AaeL_AAEL003110	Hypothetical protein AaeL_AAEL003110	108881424	Q17GD7_AEDAE	2109454
AaeL_AAEL003315	Hypothetical protein AaeL_AAEL003315	108881204	Q17FS2_AEDAE	2102384
*AaeL_AAEL004390*	*hypothetical protein AaeL_AAEL004390*	*403182639*	*Q17CY7_AEDAE*	
AaeL_AAEL008137	Hypothetical protein AaeL_AAEL008137	108875879	Q16ZM2_AEDAE	2086414
AaeL_AAEL008797	Hypothetical protein AaeL_AAEL008797	108875180	Q16XN7_AEDAE	2090533
AaeL_AAEL008799	Hypothetical protein AaeL_AAEL008799	108875181	Q16XN6_AEDAE	2090892
AaeL_AAEL009599	Hypothetical protein AaeL_AAEL009599	108874303	Q16VE2_AEDAE	2110735
AaeL_AAEL014128	Hypothetical protein AaeL_AAEL014128	108869375	Q16H79_AEDAE	2092832

^*a*^Corrected; UniProtKB ID = UniProt (http://www.uniprot.org/); DAVID ID = DAVID https://david.ncifcrf.gov/).

Gene name = VectorBase (https://www.vectorbase.org/); Protein Name = NCBI; GI Number = NCBI (http://www.ncbi.nlm.nih.gov/); **No DAVID ID available**; *Duplicate*.

#### Ovary Metal-Associated Proteins Expressed PBM

**Fig. 2.**
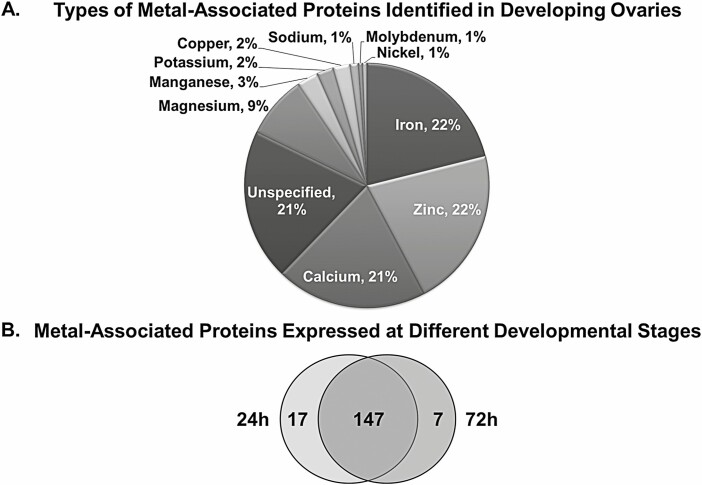
**A. Types of Metal-Associated Proteins Identified in *Ae. aegypti* Developing Ovaries.** Proteins identified using Scaffold were categorized using GO terms obtained as described in Fig. 1 to find metal-associated proteins. Metal-associated protein categories were expressed as a percentage of the total number of metal-associated proteins identified from both time points PBM. **B. Metal-Associated Proteins Expressed at Different Developmental Stages.** Metal-associated proteins detected in early development (24 hr) and late development (72 hr) PBM were identified using the stringency criteria described in the Materials and Methods. Of 171 metal-associated proteins identified using GO terms, 17 proteins were detected only at 24 hr PBM, while 7 proteins were detected only at 72 hr PBM.

The second most abundant category of PBM ovary proteins identified by GO analysis is the metal-associated proteins. These proteins represent 11% (171/1496) of the female ovary proteome, 22% have iron (38/171) or zinc (38/171) as a co-factor (Fig. 2A), and 21% (36/171) are proteins associated with calcium. Of calcium-associated proteins, 17% (6/36) are integral membrane proteins. The database of the *Ae. aegypti* ovary metal-associated proteins identified from our ovarian database is available in Supplement 5 (online only). Of the 171 *unique* proteins in this database, 17 were identified only at 24 hr PBM (Fig. 2B; Supplement 5 [online only], light grey) and 7 only at 72 hr PBM (Fig. 2B; Supplement 5 [online only], dark grey).

We also identified proteins associated with iron/heme metabolism (Table 6) and analyzed the iron/heme gene enrichment against the whole *Ae. aegypti* genome (Supplement 6 [online only]). Of the iron-binding proteins, 26% are engaged with iron-sulfur clusters (11/42). Iron sulfur clusters are required for proteins involved in numerous processes, including the electron transport chain as well as defense/detoxification (e.g., NADH-ubiquinone oxidoreductase (AaeL_AAEL005508, AaeL_AAEL012552), ABC transporter (AaeL_AAEL010059), ubiquinol-cytochrome c reductase iron-sulfur subunit (AaeL_AAEL003675), (Rouault and Maio 2017, Braymer and Lill 2017). We also found several homologs of proteins that participate in mammalian iron metabolism. We identified ferrochelatase (FECH/protoheme ferrolyase; AaeL_AAEL005415), the enzyme that catalyzes the insertion of Fe^2+^ in the terminal step in heme biosynthesis converting protoporphyrin IX into heme (Hamza and Dailey 2012); mitochondrial aconitase (mAco/ACO2: AaeL_AAEL003734), an iron-sulfur cluster enzyme involved in the regulation of cellular energy metabolism (Lushchak et al. 2014); succinate dehydrogenase (SDH: AaeL_AAEL010330), an enzyme involved in the Krebs cycle that is regulated by iron in *Drosophila* (Melefors 1996, Kim et al. 2012); and cytoplasmic aconitase (cAco/ACO1, AaeL_AAEL008216). In mammals, under adequate iron conditions, an iron sulfur cluster present in cytoplasmic aconitase gives the protein enzymatic capacity to convert citrate to isocitrate. However, in iron deficient conditions, the cluster is lost from the protein and cytoplasmic aconitase becomes the iron regulatory protein 1 (IRP1). IRP1 regulates translation of several proteins involved in iron homeostasis and was previously characterized in mosquitoes (Zhang et al. 2002, Ghosh et al. 2015, Holmes-Hampton et al. 2018). We also found a multicopper oxidase identified by GO analysis, Laccase-like multicopper oxidase 1 (AaeL_AAEL007415). In mammals, multicopper oxidases involved in iron metabolism allow ferrous to ferric conversion (Vashchenko and MacGillivray 2013). More detailed information on multicopper oxidases in insects can be found in the review by Dittmer and Kanost (2010). As expected, we identified transferrin (TF: AaeL_AAEL015458) and the ferritin subunits. Although mosquito TF binds a single iron atom, it does not serve as a major transport protein in mosquitoes (Geiser and Winzerling 2012). Available information indicates it is more likely involved in mosquito immunity (Yoshiga et al. 1997, Harizanova et al. 2005) and development (Puri et al. 2008). Ferritins can store up 4,500 atoms of iron (Theil 2012, Plays et al. 2021). Ferritin nanocages self-assemble from mixtures of 24 or 12 catalytically active (heavy chain [H/FTH1]) and inactive, stabilizing (light chain [L/FTL1]) subunits (Theil 2013). In mosquitoes, ferritin functions as both a major iron storage protein and an iron transporter in hemolymph from the gut to the tissues (Zhou et al. 2007, Geiser et al. 2009).

**Table 6.** *Ae. aegypti* iron/heme-associated proteins expressed in developing ovaries

Gene Name	Protein Name	GI Number^*a*^	UniProtKB ID	DAVID IDs
AaeL_AAEL010059	abc transporter, putative	108873788	Q16U05_AEDAE	2083205
AaeL_AAEL004860	Acireductone dioxygenase	108879511	Q0IFH8_AEDAE	2101138
AaeL_AAEL008216	Aconitase	108875800	Q16ZG5_AEDAE	2108492
AaeL_AAEL003734	Aconitase, mitochondrial	108880755	Q17EL3_AEDAE	2085427
AaeL_AAEL001501	Anamorsin, putative	108883178	DRE2_AEDAE	2084776
*AaeL_AAEL013407*	*Catalase*	*94468602*	*Q1HRH7_AEDAE*	
*AaeL_AAEL013407*	*Catalase*	*108870108*	*Q16J86_AEDAE*	*2095165*
AaeL_AAEL010017	Cytochrome B5 (cytb5)	108873820	Q16U40_AEDAE	2087867
AaeL_AAEL004450	Cytochrome b5, putative	108879914	Q17CQ7_AEDAE	2111253
AaeL_AAEL011871	Cytochrome C1	108871782	Q16NS5_AEDAE	2094013
AaeL_AAEL011463	Cytochrome P450	108872236	Q16Q15_AEDAE	2093905
AaeL_AAEL006827	Cytochrome P450	108877327	Q174P6_AEDAE	2105305
AaeL_AAEL004054	Cytochrome P450	108880360	Q17DS9_AEDAE	2116061
AaeL_AAEL002046	Cytochrome P450	108882552	Q17JC7_AEDAE	2115184
AaeL_AAEL005415	Ferrochelatase	108878883	Q17A56_AEDAE	2103523
*AaeL_AAEL007385*	*HCH, ferritin heavy chain-like protein*	*13195275*	*Q9NDI4_AEDAE*	
*AaeL_AAEL007385*	*HCH, ferritin subunit 1, putative*	*108876700*	*FRI_AEDAE*	*2093013*
AaeL_AAEL007383	LCH1, secreted ferritin G subunit precursor, putative	108876698	Q172H3_AEDAE	2101903
AaeL_AAEL002158	LCH2, secreted ferritin G subunit precursor, putative	108882438	Q17J46_AEDAE	2112545
AaeL_AAEL004195	membrane associated progesterone Receptor	108880248	Q0IFR2_AEDAE	2084300
AaeL_AAEL008072	NADH-plastoquinone oxidoreductase	108875970	Q16ZS6_AEDAE	2097107
AaeL_AAEL012552	NADH-ubiquinone oxidoreductase	108871047	Q16LR5_AEDAE	2099079
AaeL_AAEL005508	NADH-ubiquinone oxidoreductase 24 kDa subunit	108878788	Q1HRL6_AEDAE	2104643
AaeL_AAEL007681	NADH-ubiquinone oxidoreductase flavoprotein 1 (ndufv1)	108876370	Q171D1_AEDAE	2115758
AaeL_AAEL003349	Nadph cytochrome P450	108881172	Q17FM7_AEDAE	2108937
AaeL_AAEL013171	Oxidase/peroxidase	108870372	Q16JY6_AEDAE	2108302
AaeL_AAEL004401	Peroxinectin	403182638	Q17CY9_AEDAE	2107800
*AaeL_AAEL004390*	*Peroxinectin*	*403182639*	*Q17CY7_AEDAE*	*2105413*
AaeL_AAEL004386	Peroxinectin	108879994	PERC_AEDAE	2091458
AaeL_AAEL003612	Peroxinectin	108880850	Q17EY4_AEDAE	2107934
**AaeL_AAEL017029**	**Phenylalanine hydroxylase**	**27461242**	**Q8I901_AEDAE**	
AaeL_AAEL008099	Procollagen-lysine,2-oxoglutarate 5-dioxygenase	108875910	Q0IER9_AEDAE	2094725
AaeL_AAEL010330	Succinate dehydrogenase	108873481	Q16TA7_AEDAE	2099325
AaeL_AAEL015458	Transferrin	108867744	Q1DGV7_AEDAE	2112883
*AaeL_AAEL003675*	*Ubiquinol-cytochrome c reductase iron-sulfur subunit*	*403182596*	*J9HSC0_AEDAE*	*2090557*
*AaeL_AAEL003675*	*ubiquinol-cytochrome c reductase iron-sulfur subunit*	*108880827*	*Q17EQ1_AEDAE*	
AaeL_AAEL002912	wd-repeat protein	108881643	CIAO1_AEDAE	2099845
AaeL_AAEL004234	Conserved hypothetical protein	108880166	Q17DE6_AEDAE	2097137
AaeL_AAEL001185	Conserved hypothetical protein	108883502	Q17M17_AEDAE	2084212
AaeL_AAEL000507	Conserved hypothetical protein	108884221	Q17P51_AEDAE	2090052
AaeL_AAEL000496	Conserved hypothetical protein	108884222	Q17P50_AEDAE	2093466
*AaeL_AAEL004390*	*Hypothetical protein AaeL_AAEL004390*	*403182639*	*Q17CY7_AEDAE*	

Gene name = VectorBase (https://www.vectorbase.org/); Protein Name = NCBI; GI Number = NCBI (http://www.ncbi.nlm.nih.gov/);**No DAVID ID available**; *Duplicate*; Light Grey = 24 hr; Dark Grey = 72 hr.

^*a*^Corrected; UniProtKB ID = UniProt (http://www.uniprot.org/); DAVID ID = DAVID (https://david.ncifcrf.gov/).

We anticipated identifying the HCH (AaeL_AAEL007385) and LCH1 (AaeL_AAEL007383) ferritin subunits; surprisingly, we also found a second ferritin light chain homologue (LCH2, AaeL_AAEL002158) expressed at both development stages PBM. We empirically evaluated this subunit and found that LCH2 differs from all other ferritin subunits in that it is unresponsive to iron exposure. We reported high levels of LCH2 message and LCH2 protein in ovaries of *Ae. aegypti* (Geiser et al. 2017). Despite numerous studies on mosquito ferritin, the LCH2 had not been previously identified, perhaps, because available antiserums failed to react with LCH2 and the mass of LCH2 is similar to that of the other ferritin subunits.

**Fig. 3.**
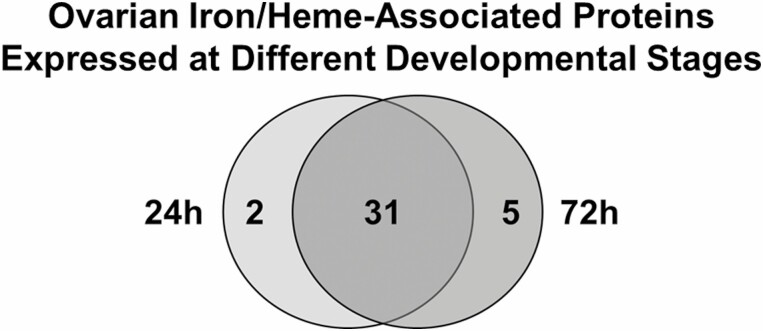
***Ae. aegypti* Ovary Iron/Heme-Associated Proteins Expressed at Different Developmental Stages.** Scaffold identified proteins were categorized using GO terms obtained as described in Fig. 1 to find iron/heme-associated proteins. Proteins detected in early development (24 hr) and late development (72 hr) PBM were identified using the stringency criteria described in the Materials and Methods. Of 38 iron/heme-associated proteins identified using GO terms, 2 proteins were detected only at 24 hr PBM, while 5 proteins were detected only at 72 hr PBM.

Some mosquito iron/heme-associated proteins are differentially expressed PBM by developmental stage (Fig. 3); 2 proteins were detected only at the early stage (Table 6, light grey) and 5 proteins were detected only at the later stage (Table 6, dark grey). Procollagen-lysine,2-oxoglutarate 5-dioxygenase (AaeL_AAEL008099), an orthologue to *Drosophila Procollagen lysyl hydroxylase* (*Plod*) that was recently shown to contribute to egg elongation in flies (Jha et al. 2015), was found only at 24 hr PBM, as was one of the four cytochrome P450s, AaeL_AAEL004054. A different cytochrome P450, AaeL_AAEL002046, was detected only at 72 hr PBM. In *Ae. aegypti*, cytochrome P450s are a family of 160 proteins that act on paired donors with incorporation or reduction of molecular oxygen (Stevenson et al. 2012) participating in heme and iron binding, oxidoreductase activity, and monooxygenase activity. AaeL_AAEL004054 gene expression is upregulated by nitroquine treatment, an antimalarial drug, in *An. stephensi* [Liston (Diptera: Culicidae); Indo-Pakistan malaria mosquito] (Zhang et al. 2014), while elevated levels of AaeL_AAEL002046 gene expression has been shown in pyrethroid resistant *Ae. aegypti* populations (Stevenson et al. 2012). Two peroxinectins also were detected only at 72 hr PBM (AaeL_AAEL004401 and AaeL_AAEL003612). These are orthologous to the *Drosophila* peroxinectin-like gene (*Dpxt*) that is expressed at high levels in late oogenesis, and may be involved in microbicidal and apoptotic cell phagocytosis as well as the cell adhesion processes (Vazquez et al. 2002). Two conserved hypothetical proteins, AaeL_AAEL000496 and AaeL_AAEL000507, also are present only at 72 hr PBM, and are predicted to have peroxidase activity, bind heme, and respond to oxidative stress.

The other conserved hypothetical proteins present at both time points PBM are predicted to be involved in oxidation-reduction (AaeL_AAEL001185) and in iron-sulfur cluster binding (AaeL_AAEL004234). While the only hypothetical protein in this list is a duplicate entry of the peroxinectin gene, AaeL_AAEL004390, that binds heme, participates in the response to oxidative stress and in eggshell chorion assembly, and has peroxidase activity (Vazquez et al. 2002, Schmidt et al. 2010, Sivakamavalli et al. 2016).

Notably, during our analysis, two proteins were identified from species other than *Ae. aegypti*: UniProtKB ID: Q5QIR4 and UniProtKB ID: D3JAI6. Both are from the *Wolbachia* endosymbionts, which is a maternally inherited intracellular bacterial symbiont that has been shown to regulate iron metabolism and fecundity in insects (Kremer et al. 2009, Gill et al. 2014).

#### Comparison of Our Shotgun Ovarian Proteome Database with Other Relevant Published Databases

Ovarian proteins, including ferritin, could come from hemolymph, tissue synthesis, or both. Paskewitz and Shi (2005) reported ferritin heavy and light chain1 subunits in a proteomic study of hemolymph proteins from *An. gambiae* [Giles (Diptera: Culicidae); African malaria mosquito]. The presence of ferritin in hemolymph and transcripts for all three subunits in ovarian tissues of *Ae. aegypti* has been documented previously (Geiser et al. 2017). When we compared our protein database to that provided by Paskewitz and Shi using the protein name as an identifier, we also found putative matches for apoLP-III, aldo/keto reductase, phosphoglyceromutase, triosephosphate isomerase, and adenylate kinase.

Due to limited resources, we were able to analyze only two development stages and selected those that we thought would be most likely to express the greatest numbers of different proteins. We did not evaluate proteins expressed by ovaries before blood feeding. However, Dias-Lopes et al. (2016) identified proteins in the reproductive tract of sugar-fed *An. aquasalis* [Curry (Diptera: Culicidae); South and Central American malaria mosquito]. We evaluated our database for the presence of the proteins in the Dias-Lopes et al. database using the protein name/description as provided in their reported data as an identifier (Online Resource 1 [Dias-Lopes et al. 2016]). We included their ‘identified’ and ‘putative’ proteins, however, excluded their ‘uncharacterized’ proteins and proteins identified with only one peptide. Of more than 600 proteins, we found 403 (67%) with a potential match in our database, 250 identified proteins and 153 putative proteins (Supplement 7 [online only]). Thus, it is likely that these proteins are expressed in ovaries before blood feeding, as well as during oogenesis. Indeed, Dias-Lopes et al. (2016) reported identifying nine proteins involved in oogenesis expressed in the sugar-fed female including ferritin HCH, ferritin LCH1, and transferrin. The remaining proteins in the Dias-Lopes et al. database could not be matched by protein name or description to proteins in our database. A further analysis showed that, of the iron-associated proteins we identified using shotgun proteomics, only ferritin HCH, ferritin LCH1, and transferrin were present in the Dias-Lopes et al. database. This would suggest shotgun proteomics successfully detected several iron-associated proteins with increased expression during development, and that these proteins when further evaluated could help to complete the picture of iron metabolism in these animals. Sreenivasamurthy et al. (2018) also reported a comparison of proteins expressed by several tissues including the ovary in sugar-fed *An. stephensi,* the reader is referred to this source for further information on proteins expressed before blood feeding.

Marinotti, et al. (2014) used shotgun proteomics to evaluate the eggshell proteome from *Ae. aegypti* ovaries. They obtained the eggs by dissection at 72 hr PBM and extracted only the eggshell proteins. Of the 127 proteins they reported, we found 51% matching proteins in our database. We might expect a lowered matching with the eggshell proteome, as we did not obtain the eggshell proteins by a separate dissection and extraction.

Akbari et al. (2014) reported the transcriptome for *Ae. aegypti* ovaries for several developmental time points (nonblood fed, 12, 24, 36, 48, 60, and 72 hr PBM). We compared our protein database to their gene database using the short gene name as an identifier. Using annotated genes only, we found matching transcripts for 99.4% of the proteins in our database (Supplement 8 [online only]). Of the proteins we identified only at the early development stage, 60.7% of the transcript matches showed the greatest transcript expression by or before 36 hr, while for proteins expressed only at the later stage, 68% of transcripts reached the greatest expression at 48 hr or later. We also identified transcripts for 100% of the proteins identified as iron-associated in our ovarian proteome database. Although it is beyond the scope of this project, it will be interesting to see if the proteins we identified have transcripts and/or genes with known iron or metal responsive control elements.

#### TMT-Labeling Proteomics Analysis of Proteins Expressed on Different Diets

At the time we obtained our data, methodology for quantitating shotgun proteomic data were not available. To obtain some quantitative data on ovarian protein expression in *Ae. aegypti*, we evaluated proteins expressed in response to an iron-controlled meal using proteomics with TMT-labeling. Due to limited resources, we selected to analyze proteins expressed only during the early developmental stage (24 hr PBM). The two runs (each in triplicate) identified 134 proteins (Tube A) and 96 proteins (Tube B) for a total of 230 proteins (Supplement 9 [online only]). Of these 230 proteins, 88 proteins were identical between runs and 65 of the identical proteins showed significantly different expression on the varied diets. A comparison of the TMT-labeled protein database with our shotgun ovarian database showed of the 65 differentially expressed proteins 47 were present. Why our subset of TMT-labeled proteins did not exactly match to the shotgun proteomic database we do not know. It is likely that proteins expressed on the artificial diets differed from those expressed following blood feeding as of the 18 proteins that did not match, 15 showed greater expression on the artificial diets than for animals fed on blood. Alternatively, the great differences in how the samples were prepared for analysis could account for a substantial amount of variation. For the shotgun proteomics experiments all samples were separated on gels, digested in-gel, and the individual gel slices analyzed, and then the data combined for each sample. Whereas the TMT-label experiments were from artificial bloodmeal and Porcine BM samples, trypsin digested in solution, isobaric labeled for each individual diet, and then combined for the analysis.

Twenty-nine of the proteins with significantly different expression (45%, Table 7, (-)Fe>(+)Fe) showed greater expression on the (-)Fe diet than on the (+)Fe diet, and of these, 23 (79%) showed greatest expression on (-)Fe diet relative to either the (+)Fe diet or the blood meal ((-)Fe>Porcine BM). Nineteen of these 29 proteins (65%, Table 7, grey shaded) are involved in ribosomal structure or function, including AAEL001420-PA (putative kinase involved in RNA translation), AAEL010097-PA (role in mRNA localization during oogenesis [Akbari et al. 2014]), Elongation factor 1-alpha (transfer of aminoacyl tRNAs to the ribosome [Sasikumar et al. 2012]) and RNA helicase. Among the remaining proteins, 14-3-3 protein is from a family of regulatory proteins found in most species that are essential components of phosphorylation-mediated signaling (Aitken 2006, Tinti et al. 2012), while reduced heat shock cognate 70 has been shown to permit cell proliferation (Miller and Fort 2018). AAEL011197-PA and AAEL005097-PA are two predicted members of the actin family.

**Table 7.** Differential expression of ovary proteins from *Ae. aegypti* fed an artificial bloodmeal with iron, an artificial bloodmeal without iron, or porcine bloodmeal

UniProtKB ID	Protein Names	Expression Profile
Q1HQX9	Mitochondrial phosphate carrier protein	*(-)Fe>(+)Fe*
Q1HRP2	60S ribosomal protein L22 (AAEL007771-PB)	*(-)Fe>(+)Fe*
Q1HRQ2	AAEL013069-PA (Activated protein kinase C receptor) (Guanine nucleotide-binding protein subunit beta-like protein)	*(-)Fe>(+)Fe*
Q1HRQ7	ATP synthase subunit alpha	*(-)Fe>(+)Fe*
Q1HRQ9	40S ribosomal protein S8	*(-)Fe>(+)Fe*
Q4F6X0	40S ribosomal protein S3 (AAEL008192-PA) (Ribosomal protein S3)	*(-)Fe>(+)Fe*
Q16QZ7	14-3-3 protein sigma, gamma, zeta, beta/alpha (AAEL011116-PA) (AAEL011116-PB) (AAEL011116-PC)	*(-)Fe>(+)Fe*; *(-)Fe>Porcine BM*
Q17B41	AAEL005097-PA	(-)Fe>(+)Fe; (-)Fe>Porcine BM
Q1HR24	40S ribosomal protein S14	*(-)Fe>(+)Fe*; *(-)Fe>Porcine BM*
Q1HR35	60S ribosomal protein L30	*(-)Fe>(+)Fe*; (-)Fe>Porcine BM
Q1HR99	60S acidic ribosomal protein P0	*(-)Fe>(+)Fe*; *(-)Fe>Porcine BM*
Q1HRJ3	Ribosomal protein	*(-)Fe>(+)Fe*; *(-)Fe>Porcine BM*
Q1HRT9	40S ribosomal protein S5 (AAEL013625-PA) (AAEL013625-PB) (AAEL013625-PC) (AAEL013625-PD) (Ribosomal protein S5)	(-)Fe>(+)Fe; *(-)Fe>Porcine BM*
Q1HRV1	40S ribosomal protein S2	*(-)Fe>(+)Fe*; *(-)Fe>Porcine BM*
A0A6I8T3I8	Histone H4	(-)Fe>(+)Fe; (-)Fe>Porcine BM; ***(+)Fe>Porcine BM***
A0A6I8TRR8	Uncharacterized protein	(*-)Fe>(+)Fe*; (-)Fe>Porcine BM; **(+)Fe>Porcine BM**
A0A6I8TYT7	Histone H2A	(*-)Fe>(+)Fe*; (-)Fe>Porcine BM; ***(+)Fe>Porcine BM***
Q16FA5	Heat shock protein 83	(-)Fe>(+)Fe; (-)Fe>Porcine BM; ***(+)Fe>Porcine BM***
Q16Q18	60S ribosomal protein L14	(*-)Fe>(+)Fe*; (-)Fe>Porcine BM; ***(+)Fe>Porcine BM***
Q16QR7	AAEL011197-PA (AAEL011197-PB)	(-)Fe>(+)Fe; (-)Fe>Porcine BM; **(+)Fe>Porcine BM**
Q16TW3	AAEL010097-PA	(*-)Fe>(+)Fe*; (-)Fe>Porcine BM; ***(+)Fe>Porcine BM***
Q17LC2	AAEL001420-PA (Leucine-rich immune protein [Short])	(*-)Fe>(+)Fe*; (-)Fe>Porcine BM; **(+)Fe>Porcine BM**
Q17N60	60S ribosomal protein L35a	(*-)Fe>(+)Fe*; (-)Fe>Porcine BM; ***(+)Fe>Porcine BM***
Q1HQZ5	Heat shock cognate 70	(-)Fe>(+)Fe; (-)Fe>Porcine BM; ***(+)Fe>Porcine BM***
Q1HR65	60S ribosomal protein L17	(-)Fe>(+)Fe; (-)Fe>Porcine BM; ***(+)Fe>Porcine BM***
Q1HR88	Elongation factor 1-alpha	(*-)Fe>(+)Fe*; (-)Fe>Porcine BM; ***(+)Fe>Porcine BM***
Q1HRL8	40S ribosomal protein S18	( *-)Fe>(+)Fe*; *(-)Fe>Porcine BM*; ***(+)Fe>Porcine BM***
Q1HRP0	60S ribosomal protein L27a	( *-)Fe>(+)Fe*; *(-)Fe>Porcine BM*; ***(+)Fe>Porcine BM***
Q3ZDP2	RNA helicase (EC 3.6.4.13)	*(-)Fe>(+)Fe*; *(-)Fe>Porcine BM*; ***(+)Fe>Porcine BM***
P62251	40S ribosomal protein S16	*(-)Fe>Porcine BM*
Q0IFN2	AAEL004500-PA (AAEL004500-PB)	*(-)Fe>Porcine BM*
**UniProtKB ID**	**Protein Names**	**Expression Profile**
Q1HR57	AAEL001872-PA (Mitochondrial porin) (Voltage-dependent anion channel) (Fragment)	*(-)Fe>Porcine BM*
Q1HRM9	60S acidic ribosomal protein P2	*(-)Fe>Porcine BM*
Q1HRN4	60S ribosomal protein L21	*(-)Fe>Porcine BM*
J9HFM9	AAEL017516-PB	(-)Fe>Porcine BM; **(+*)Fe>(-)Fe***; **(+)Fe>Porcine BM**
A0A0N8ES04	Ribosomal protein L15	*(-)Fe>Porcine BM;* ***(+)Fe>Porcine BM***
A0A6I8TK86	Uncharacterized protein	(-)Fe>Porcine BM; ***(+)Fe>Porcine BM***
A0A6I8U458	Uncharacterized protein	(-)Fe>Porcine BM; **(+)Fe>Porcine BM**
Q16FB1	60S ribosomal protein L27	*(-)Fe>Porcine BM;* ***(+)Fe>Porcine BM***
Q16PM9	AAEL011584-PA (Chaperonin-60kD, ch60)	(-)Fe>Porcine BM; ***(+)Fe>Porcine BM***
Q16ZH3	60S ribosomal protein L6	*(-)Fe>Porcine BM;* ***(+)Fe>Porcine BM***
Q174U3	60S ribosomal protein L18a	*(-)Fe>Porcine BM;* ***(+)Fe>Porcine BM***
Q179S9	AAEL005515-PF (Heterogeneous nuclear ribonucleoprotein)	*(-)Fe>Porcine BM;* ***(+)Fe>Porcine BM***
Q17Q32	Enolase-phosphatase E1 (EC 3.1.3.77) (2,3-diketo-5-methylthio-1-phosphopentane phosphatase)	*(-)Fe>Porcine BM;* ***(+)Fe>Porcine BM***
Q1HQJ0	60S ribosomal protein L4 (AAEL009994-PA) (Ribosomal protein L4)	*(-)Fe>Porcine BM;* ***(+)Fe>Porcine BM***
Q1HR32	60S ribosomal protein L8	*(-)Fe>Porcine BM;* ***(+)Fe>Porcine BM***
Q1HR53	Tubulin alpha chain	(-)Fe>Porcine BM; ***(+)Fe>Porcine BM***
Q1HR63	60S ribosomal protein L13a (Fragment)	(-)Fe>Porcine BM; **(+)Fe>Porcine BM**
Q1HR82	RNA helicase (EC 3.6.4.13)	*(-)Fe>Porcine BM;* ***(+)Fe>Porcine BM***
Q1HR85	60S ribosomal protein L24 (AAEL008329-PA) (Ribosomal protein L24)	(-)Fe>Porcine BM; **(+)Fe>Porcine BM**
Q1HRJ1	60S ribosomal protein L28	*(-)Fe>Porcine BM;* ***(+)Fe>Porcine BM***
Q1HRN0	60S ribosomal protein L44 (60S ribosomal protein L44 L41, putative) (AAEL003942-PA)	*(-)Fe>Porcine BM*; **(+)Fe>Porcine BM**
Q5QC94	40S ribosomal protein S13	*(-)Fe>Porcine BM;* ***(+)Fe>Porcine BM***
Q6Q9G1	Ribosomal protein L37	(-)Fe>Porcine BM; **(+)Fe>Porcine BM**
Q1HR81	60S ribosomal protein L7 (AAEL012585-PA) (AAEL012585-PB) (Ribosomal protein L7)	**(+*)Fe>(-)Fe***; ***(+)Fe>Porcine BM***
A0A1S4FCM3	Uncharacterized protein	***(+)Fe>(-)Fe***; Porcine BM>(+)Fe
Q16927	Vitellogenin-A1 (VG) (PVG1) [Cleaved into: Vitellin light chain (VL); Vitellin heavy chain (VH)]	**(+)Fe>(-)Fe**; Porcine BM>(+)Fe; Porcine BM>(-)Fe
Q17AX5	AAEL005170-PA	***(+)Fe>Porcine BM***
Q1HRI6	60S ribosomal protein L12	***(+)Fe>Porcine BM***
Q16V93	AAEL009642-PA (Cathepsin b)	Porcine BM>(-)Fe
Q1HR72	60S ribosomal protein L9	*Porcine BM>(+)Fe*
Q1HRS6	40S ribosomal protein S4	*Porcine BM>(+)Fe*
J9HEW1	AAEL017501-PA	*Porcine BM>(+)Fe*; *Porcine BM>(-)Fe*
Q177I2	AAEL006138-PA	Porcine BM>(+)Fe; Porcine BM>(-)Fe
Q1HR28	40S ribosomal protein S28	*Porcine BM>(+)Fe*; *Porcine BM>(-)Fe*

Difference between increased or decreased expression of a protein due dietary iron was significant (*p*<0.05) using the Holm-Sidak method for multiple unpaired *t*-tests in Prism 6 as described in the Materials and Methods. UniProtKB ID = UniProt (http://www.uniprot.org/);**Boldfaced** = Increased expression on the (+)Fe diet; Grey shaded = Involved in ribosomal structure or function; Underlined = Significantly different (SD) expression for both runs performed in triplicate; *Italics* = SD expression for one run performed in triplicate; (+)Fe = ABM with Iron; (-)Fe = ABM without Iron; Porcine BM = Porcine bloodmeal.

Only four proteins (Table 7, boldfaced, (+)Fe>(-)Fe) were identified with a significantly increased expression on the (+)Fe diet relative to Fe(-) diet. These included: vitellogenin-A1, a protein synthesized in fat body and imported into the ovaries during egg formation, as well as AAEL017516-PB and 60S ribosomal protein L7, both constituents of the ribosome.

A comparison of the expression of proteins from (+)Fe fed animals with those of Porcine blood-fed animals identified 38 (Table 7, boldfaced, (+)Fe>Porcine BM) with a greater expression on the (+)Fe diet. These included 25 proteins involved in ribosomal structure or function, as well as, Tubulin alpha chain, Enolase-phosphatase E1, AAEL005170-PA (putative cytochrome c oxidase subunit iv, VectorBase), AAEL005515-PF (heterogeneous nuclear ribonucleoprotein), and AAEL011584-PA (putative 60 kDa heat shock protein).

Seven proteins showed significantly greater expression for the Porcine BM relative to the (+)Fe diet (Table 7, Porcine BM>(+)Fe). These included three ribosomal proteins, Vitellogenin-A1, and a putative vitellogenin (AAEL006138-PA).

A comparison of proteins from animals fed (-)Fe with Porcine blood-fed animals yielded 48 proteins that showed greater expression on the (-)Fe diet than for Porcine BM (Table 7, (-)Fe>Porcine BM). Thirty-three of these proteins (69%, grey shaded) are involved in ribosomal function and structure—including AAEL004500-PA, a putative translation elongation factor. Of the remaining proteins, AAEL001872-PA is a putative voltage-dependent anion channel (VDAC) mitochondrial porin. Mitochondrial porins function in the outer membrane of the mitochondria to efficiently import carrier precursors (Ellenrieder et al. 2019). An interaction of a VDAC with tubulin has been shown to control mitochondrial metabolism in mammalian cells (Fang and Maldonado 2018). AAEL009642-PA (Cathepsin b), one of only five proteins to demonstrate a significant decrease in the expression on the (-)Fe diet compared to the Porcine BM, was identified in the recent paper by Martins et al. (2021) examining proteins of the head and salivary glands of *Ae. aegypti*.

### Conclusions

We used proteomic analysis to identify proteins in the developing ovaries of *Ae. aegypti* to provide further insight into mosquito oogenesis and on the expression of iron-associated proteins. As far as we can determine ours is the first report of proteins expressed in this tissue following a bloodmeal and the first to report protein expression at two developmental stages of oogenesis. Our database matches well with the transcript database of Akbari et al. (2014), confirms the expression of the proteins for numerous transcripts they identified and can provide some insight into the timing of translation for proteins expressed in specific developmental stages. Our database also can serve as a platform for others engaged in seeking candidates for interference in the development process of these animals. It is clear from the numbers of hypothetical proteins found by our work that the function of many proteins involved in oogenesis and egg development remains to be elucidated.

We show that metal-associated proteins play an important role in *Ae. aegypti* oogenesis. In fertilized chicken eggs, it was demonstrated that the yolk is the major source of the minerals, such as Mn, P, Fe, Ca, Cu, and Zn that are essential for early, as well as later, embryonic development (Yair and Uni 2011). Significantly, iron-associated proteins make up 22% of the identified metal-associated proteins in *Ae. aegypti* ovaries. Iron is required for developing mammals (Gambling et al. 2011, Lipinski et al. 2013), and several minerals, including iron, require protein association for storage, transport, and utilization. Little is known about iron movement in *Ae. aegypti* cells. In other species, iron movement inside cells requires chaperone proteins and membrane iron transporters. It remains to be determined if the putative ABC transporter and cytochrome B5 we identified might serve in these processes.

We identified several proteins engaged in the binding of or the formation of iron sulfur clusters implying these processes are necessary for development in mosquitoes similar to that reported for *Drosophila* (Marelja et al. 2018). We also identified ferrochelatase, and several proteins known to bind heme in other species. The importance of heme was demonstrated in in *Schistosoma mansoni* [Larue (Diplostomida: Schistosomatidae); human blood fluke] where inhibition of heme uptake significantly reduced egg production and retarded development (Toh et al. 2015). The importance of iron for oogenesis also was demonstrated in *Ixodes ricinus* [Linnaeus (Ixodida: Ixodidae); castor bean tick], another blood feeding arthropod (Hajdusek et al. 2009), where RNA silencing of ferritin reduced hatch rate. Iron/heme proteins also are essential for the metabolic response to oxidative stress, iron homeostasis, heme biosynthesis, eggshell chorion assembly, and energy production.

Importantly, through this work we identified the LCH2, and subsequently obtained empirical data supporting the expression of LCH2 in ovaries and its lack of control by iron (Geiser et al. 2017). Why this species expresses a second LCH subunit is not known. Mammalian ferritin is composed of 24 subunits of two types, heavy chain or light chain, and protein isoforms reflect the numbers of each type of subunit present in the molecule (Liu and Theil 2005). So, for example, the storage isoform consists of more light chain subunits than heavy chain subunits. X-ray crystal structure of insect ferritin shows 12 heavy chain subunits and 12 light chain subunits (Hamburger et al. 2005). Thus, we suggest that insects might substitute the different light chain subunits (LCH1 and LCH2) to form ferritin isoforms in these animals. If this is the case, it seems probable that other LCH subunits remain to be found as cDNAs and multiple genes for LCH ferritin subunits in mosquitoes have been reported (Dunkov and Georgieva 2006).

To obtain quantitative data, we employed TMT-labeling of proteins expressed by animals fed an iron-controlled diets. The differentially expressed proteins we identified using this technique were obtained with high confidence in the expression differences according to the criteria defined in the Materials and Methods. Differential expression suggests that iron could be involved in the regulation of expression of some of the proteins we identified either by repressing expression for proteins with the greatest expression on the (-)Fe diet relative to the (+)Fe diet, or by enhancing expression of proteins that showed the greatest expression on the (+)Fe diet relative to that of the (-)Fe diet or the bloodmeal.

Why several proteins involved in ribosomal structure, function, and translation processes show greater expression for animals fed the artificial diets than for animals fed the Porcine BM, we do not know. Given that the ribosomal apparatus is present following blood feeding as shown by the shotgun proteomics analysis and that vitellogenin (a protein imported from fat body into the ovaries) shows the greatest levels in the Porcine blood-fed animals, we speculate that the processes of oogenesis that involve protein synthesis by ovaries vary with time during egg development and in blood-fed animals are reduced by 24 hr relative to earlier time intervals. This suggests that the rate of development for animals fed artificial diets lags that of blood-fed animals. The bloodmeal contains many constituents including numerous nutrients and signaling factors not found in the artificial meals. Possibly some of these factors increase the rate of ovarian and egg development in blood-fed animals. If this is the case, then rate of development would be an important factor to consider when comparing results from animals fed artificial diets with those fed on blood.

In summary, we used two very different approaches to identify potential proteins involved in oogenesis with differential expression associated with iron metabolism. The data collected from the shotgun proteomics analysis identified 1496 proteins including 38 with a known or a potential relationship to iron metabolism. The fitness of this approach has been demonstrated by empirical evaluation of LCH2. The second study showed isobaric labeling could detect differential expression of proteins for animals fed two types of artificial diets varying in one nutrient and identified some candidates that showed expression control by iron. The further usefulness of this technique remains to be seen as identified proteins are explored for their roles in iron metabolism.

Since we conducted these analyses, others have shown that isobaric labeling can be coupled with shotgun proteomics to produce reliable large-scale quantification. This is important when seeking proteins involved in mineral metabolism as many could be less abundant proteins and would be missed using only isobaric labeling methods without shotgun proteomics. In support of this notion, Martins et al. (2021) recently combined the techniques of shotgun proteomics with isobaric protein labeling and reported results for the proteome from heads and salivary glands of *Ae. aegypti* infected with the *Wolbachia* wMel strain and ZIKV. They recovered more than 4,000 proteins. Their work as well as that of others (De Mandal et al. 2020) supports the power and the recommendation of combining these two methods in future studies with similar goals.

## Supplementary Material

ieac018_suppl_Supplementary_Materials
