# Supplementary material for: Shotgun and TMT-Labeled Proteomic Analysis of the Ovarian Proteins of an Insect Vector, Aedes aegypti (Diptera: Culicidae)
Source: J Insect Sci. 2022 Mar 18;22(2):7. doi: 10.1093/jisesa/ieac018 (PMC8932505; doi:10.1093/jisesa/ieac018)
Supplement: ieac018_suppl_Supplementary_Materials [file ieac018_suppl_supplementary_materials.zip › ieac018_suppl_Supplement 2.docx]

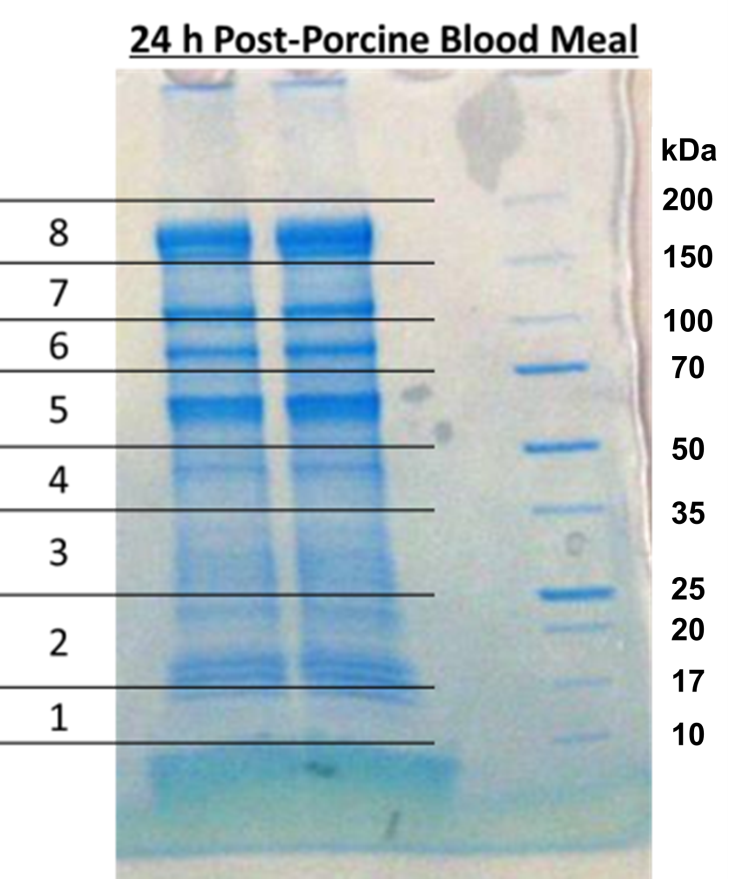


**Supplement 2. Separation of *Ae. aegypti* 24 h PBM Ovary/Egg Proteins.** Samples were prepared as described in the Materials and Methods*.* Proteins were separated via 12% SDS-PAGE and stained with Bio-Safe Coomassie Blue. Each gel lane was cut into 8 gel slices according to molecular weight as indicated.
